# The Distribution of Standard Deviations Applied to High Throughput Screening

**DOI:** 10.1038/s41598-018-36722-4

**Published:** 2019-02-04

**Authors:** Quentin S. Hanley

**Affiliations:** School of Science and Technology, Nottingham Trent University Clifton Lane, Nottingham, NG11 8NS United Kingdom

## Abstract

High throughput screening (HTS) assesses compound libraries for “activity” using target assays. A subset of HTS data contains a large number of sample measurements replicated a small number of times providing an opportunity to introduce the distribution of standard deviations (DSD). Applying the DSD to some HTS data sets revealed signs of bias in some of the data and discovered a sub-population of compounds exhibiting high variability which may be difficult to screen. In the data examined, 21% of 1189 such compounds were pan-assay interference compounds. This proportion reached 57% for the most closely related compounds within the sub-population. Using the DSD, large HTS data sets can be modelled in many cases as two distributions: a large group of nearly normally distributed “inactive” compounds and a residual distribution of “active” compounds. The latter were not normally distributed, overlapped inactive distributions – on both sides –, and were larger than typically assumed. As such, a large number of compounds are being misclassified as “inactive” or are invisible to current methods which could become the next generation of drugs. Although applied here to HTS, it is applicable to data sets with a large number of samples measured a small number of times.

## Introduction

One of the most important measurements currently made is the assessment of the “activity” of drug candidates toward an assay target. These measurements have been made millions of times as part of routine high throughput screening (HTS) activities for drug discovery. In a now classic paper, Zhang, Chung and Oldenburg^1^ provided the Z and Z′ metrics for assessing assay suitability for HTS. The general approach articulated in this paper has remained a cornerstone of thinking in HTS and there have been a number of excellent subsequent papers advancing practices for identifying active compounds^2–5^. Zhang *et al*. assumed most compounds have “little or no activity”. This means that a histogram of screening measurements should show a main distribution having a width representing measurement error and a small number of “active” compounds. Accepting this means that the position of a particular measurement of compound activity within its distribution is almost never meaningful. Unlike the distribution of human heights^6^ where each height measurement meaningfully places an individual into a useful position within the overall distribution, HTS cannot do this except in extreme cases.

A range of procedures have been described to control spatial effects in plates^2,5,7,8^, and further guidance exists for many aspects of hit detection^7,9^. Presentation of the problem of HTS as discriminating compounds into a distribution called “inactive” and another called “active” can be found in many papers on HTS often illustrated with two normal distributions^1,2,9^. The usual procedure for assigning a compound as “active” is by consideration of mean or median values (for *N* > 1) or by comparison of single measurements with a mean or median value (*N* = 1). This is, however, only one of many valid ways to make a statistical inference. Detection of differentness can also be done by comparison of variance^10^. To do either effectively, measurement error must be well understood. In addition, use of screening data in cheminformatics^11,12^ requires methods that detect active compounds well or presents their probability of being active clearly. Importantly, there may be no “active” distribution, it may be several distributions, and it may be fundamentally different in shape and characteristics from the “inactive” distribution.

In primary screens, it is not uncommon for measurements of the responses generated by candidate molecules to be made only one or two times. In publicly released data sets where two replicate measurements were made, “reproducibility values” are often presented representing the correlation cosine between paired replicate measurements and the vector [1, 1]^4,13,14^. This information is sometimes used to decide whether a compound is “active”^13^. For this to be a valid metric, the histogram of such values should be compared to expectations from the distribution of “inactive” compounds.

Statistical procedures do not classify single compounds as “active”. Rather they place compounds into groups based on difference from some proposed model. Thresholds can be used to set the estimated likelihood of a false positive to a desired value when designating a compound as “active”. A better view of this activity is to state the “inactive” model, test the validity of that model, and assign the likelihood a particular compound’s measurement conforms to that model. This can remove thresholds entirely from experimental design and presentation.

Due to a range of imprecise language, it is easy to equate a compound designated as “active” due to some statistical test with “useful lead compound”. Clear warnings may be found in the literature of “frequent hitters”^15^ and Pan-Assay Interference compoundS (PAINS)^16,17^. These compounds have a range of behaviors leading them to generate responses during multiple primary screens and without care, follow up work can result in expensive and fruitless efforts^16,18^. A range of structural motifs associated with these compounds have been identified and tools are available to test for known features^19,20^. There is also clear evidence that “undesirable” features should not dismissed out of hand^21^ and PAINS motifs have been useful in some instances^22–24^. In all studies to date, there are no direct measures that might identify PAINS and promiscuous compounds from single screens. Problematic compounds have been inferred from cross referencing multiple studies.

The distribution of standard deviations^25,26^ (DSD) is a useful framework for understanding variability in large data sets containing a large number of samples measured *N* times. Its shape depends only on *N*. It is well described for the normal distribution and can be extended to log-normal data. In this context, the DSD can provide evidence for the existence of a single normally distributed process generating a set of varying mean values. Combined with evidence for heteroscedasticity from fluctuation scaling methods^10,27,28^ a clearer understanding of HTS data sets can be obtained.

To better understand the behaviour of HTS, publicly available data sets were assessed using histograms, the distribution of standard deviations, fluctuation scaling, and analysis of residuals. Although it can detect high variability “active” compounds, the purpose of the study was to better understand the distribution of “inactive” compounds and the characteristics of measurement error in these HTS data sets. The goal was to develop methods to determine the standard deviation of the measurement process, the behaviour of inactive compounds, and detect signs of bias at the measurement stage where possible. This exercise also investigates whether data are consistent with normal distributions, whether they are homoscedastic, and if skewed whether this is from measurements or the behaviour of candidate compounds.

To avoid confusion, “active” will be used here to describe a group of compounds that is different from a main group of compounds based on a statistical model applied to the results of a large scale primary screen. Such “active” compounds may or may not be useful for follow up drug development.

## Theory

Screening data sets are especially amenable to good statistical analysis due to the expectation that 300,000+ measurements should converge to “true” behaviour. If the compounds represent a mix of “active” and “inactive” compounds a histogram of measured activities is insufficient to decide if measurements follow a normal distribution or to make good decisions about compound activity. Evidence of skewing of the distribution of measured activities or the existence of long tails may lead to an incorrect conclusion that the data are inconsistent with a normally distributed homoscedastic process. The “inactive” distribution may be normally distributed and all the data may be generated by a single Gaussian process despite any observed skewing. The characteristics of “inactive” compounds and the generating process are more important for deciding if a compound is likely to be active than a mean and standard deviation computed on all measured values. One approach to assess normality of the generating process is to investigate the DSD^25,26]^. The DSD is well defined for a normal distribution and can be adapted for log-normal data. This distribution can be used to assess whether a set of non-normally distributed average values are consistent with a single homoscedastic normally distributed process. This is useful in HTS because a wide range of meaningful mean values may exist but the DSD depends only on the number of replicates, *N*, used to make a single assessment of “activity”.

**Figure 1:**
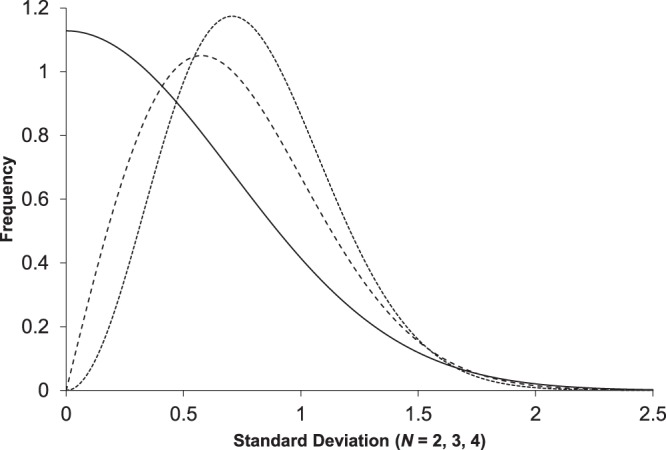
DSDs for a normally distributed data set or from a single homoscedastic normally distributed process based on three values of *N*. The curves correspond to *N* = 2 (solid line), *N* = 3 (dashed line), and *N* = 4 (dotted line).

Using the convention that ${s}_{p}=\sqrt{\frac{1}{N}\,\sum _{i=1}^{N}{({x}_{i}-\bar{x})}^{2}}$, the DSD for a given value of *N* is given by eq. 1^25,26^.

1 $${s}_{dist}({s}_{p})=2\frac{{(\frac{N}{2{\sigma }^{2}})}^{\frac{N-1}{2}}}{{\rm{\Gamma }}(\frac{1}{2}(N-1))}\,{e}^{-\frac{N{s}_{p}^{2}}{2{\sigma }^{2}}}{s}_{p}^{N-2}$$

where ${\sigma }^{2}=\frac{N{s}^{2}}{N-1}$ and Γ represents the gamma function. This family of distributions has a shape (Fig. 1) that is dependent on *N* and scaled versions of the function can be fit to a histogram from an experimental data set. This describes the expected behaviour of a data set arising from multiple trials sampling a normal distribution or a single homoscedastic normally distributed process. In the latter case, it is not necessary that the measurements themselves belong to the same normal distribution with the same value of *μ*. It is also worth noting that the expected proportion of measurements appearing with standard deviations greater than 3*s* is very small. Further, due to the maximum as standard deviation approaches 0 for *N* = 2, a high proportion of paired measurements from normally distributed processes are expected to be close together.

It is important to appreciate the relevance of this family of distributions to HTS where a large number of trials are run to sample a distribution a small number of times. Understanding the expected variability can aid interpretation of metrics routinely reported with some screens and the effect of thresholds. Examples include: the reproducibility cosine transform (RCT) which is sometimes referred to as the correlation cosine^4^ (see supplementary information (SI), section S.1) and the use of double thresholds applied to replicate measurements (see SI, section S.2).

Specifying thresholds based on 95% (or other related) expectations from a normal (or other) distribution of inactive compounds imposes an assumption that drug like compounds are rare and extreme in their activity (e.g. always distant from inactive ones). If this is not the case, arbitrarily setting criteria based on a multiple of the standard deviation will not be as effective as the ratio of all compounds within an interval to the number expected based on a model of the distribution of inactive compounds.

Not all measurement systems exhibit homoscedasticity. Indeed, such systems are rare. Fluctuation scaling methods^10,27,28^ can be used to assess this and understand the relationship between mean and standard deviation. Many measurements show heteroscedasticity and some exhibit standard deviations that scale with the mean following power laws:

2 $$s=\beta {\bar{x}}^{\alpha }$$

where *α* and *β* are constants. For a homoscedastic process that can generate many different mean values, *α* = 0. For heteroscedastic processes, *α* ≠ 0. Due to the range of data transformations applied, the relatively low number of replicates, and the existence of negative numbers in HTS, meaningful fluctuation scaling plots can be difficult to construct. However, understanding heteroscedasticity is critical for making sensible decisions about “active” vs. “inactive” compounds. This is particularly true when “active” compounds are rare. This can be modeled by considering there to be two distributions of compounds, one “inactive” with a standard deviation defined by measurement error and the other “active” with a standard deviation which may or may not be equal to that of the “inactive” distribution (see SI section S.3).

In the primary screens considered here, 1,325,382 compound assays were performed with 4583 deemed “active” (~0.3%). If this is taken as the “true” fraction of “active” compounds, a compound must be 3.4*σ* away from the mean of the “inactive” distribution before it has 50% likelihood of not being an “inactive” compound in a homoscedastic system. However, if the “active” distribution has a standard deviation 10% lower than the inactive distribution, there is no interval over which “active” compounds are present with 50% likelihood unless the mean difference between “active” and “inactive” distributions exceeds 1.47*σ*. If the “active” distribution has a 10% greater standard deviation, 50% likelihood zones can appear on both sides of the “inactive” distribution. These considerations are presented in more detail in Section S.3 of the SI.

For the purposes of developing models of inactive and active compounds the following analyses were carried out. (1) The histogram of average values was constructed to see if the data appear normally distributed and to obtain means and standard deviations. (2) A best fit scaled normal distribution was fit to the data to obtain a separate value of mean and standard deviation. (3) To assess conformity of the measured values to a single normally distributed process, the histogram of standard deviations was constructed and compared to equation 1. (4) A histogram of RCT values was compared to a histogram of normally distributed random numbers having the mean and standard deviation obtained from best fit normal distributions (see SI section S.1). (5) To estimate the shape of “active” distributions, residual plots of the histogram of average values minus the best fit normal distributions were generated. (6) Where data were available for *N* = 2, 3, and 4, histograms of standard deviations were generated for comparison with the predictions of equation 1. (7) Screens were pooled to obtain a set of “high variability active” compounds which were examined to look for structural similarities.

## Results and Discussion

### The distribution of means and the DSD

**Figure 2:**
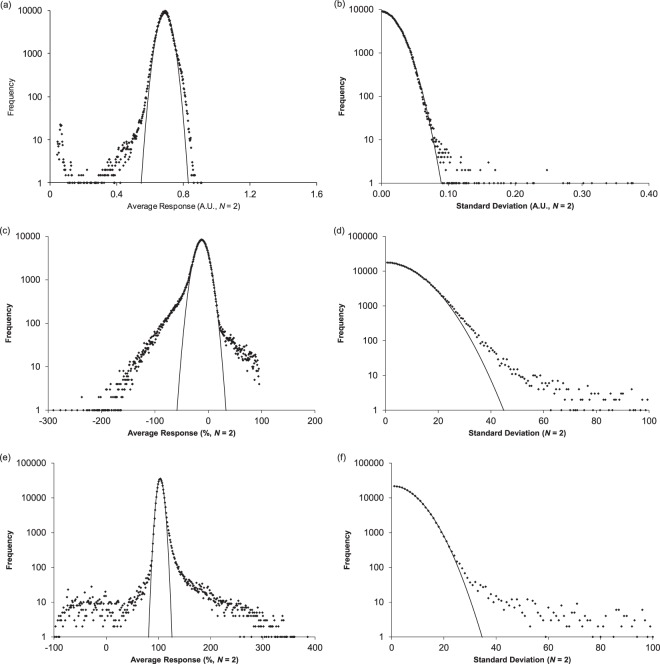
Histogram of mean values (**a**,**c**,**e**) and histogram of standard deviations (**b**,**d**,**f**) for the three screens representing 871,060 assays compounds with *N* = 2. The histogram of mean values was fit to normal distribution (solid lines in panels, a, c, and e). The histograms of standard deviations were fit to equation 1 (solid lines in panels b, d, and e). The AID1053175 data set (panels (a) and (b)) gave a poor fit to a normal distribution (**a**) but excellent correspondence to a normally distributed process (**b**). The histograms of average values from the AID488862 **(c**) and AID1224905 (**e**) data sets both exhibited some correspondence to a normal distribution with broad asymmetric tails. Correspondence to a single normally distributed process is more limited in these (**d**,**f**) than for AID1053175.

A single homoscedastic normally distributed process will generate multiple normal distributions having different mean values but the same standard deviation. The distribution of all mean values from such a process may not appear to be normally distributed but the distribution of standard deviations is expected to conform to a single normally distributed process. To test HTS data for homoscedasticity and consistency with normal statistics, the average and standard deviation were computed for each compound in three sets of HTS results and histograms constructed (Fig. 2). These included screens for inhibitors of *E. Coli* (AID 1053175, 329,176 compounds), for inhibitors of the prion protein 5′ UTR mRNA (AID488862, 335,011 compounds), and for disruptors of the interaction between Gα_i_ and GIV (AID1224905, 206,873 compounds).

**Figure 3:**
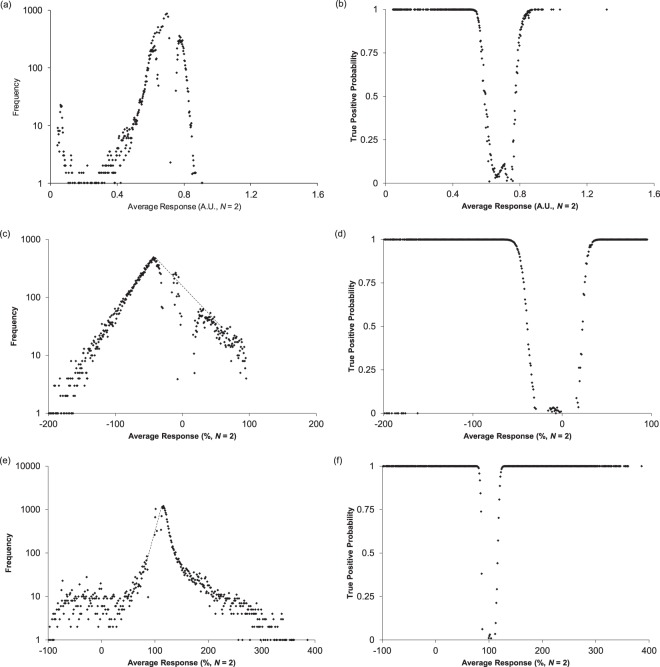
Residual histogram plots (**a**,**c**,**e**) after subtraction of the best fit normal “inactive” distributions and resulting probability plots for finding “active” compounds in a particular interval. The dotted lines in (**c**) and (**e**) are to guide the eye through a region that is probably being distorted by fitting the normal distribution to what is a more complex system. The AID1053175 data set (panels (a) and (b)) gave a residual distribution reminiscent of the original distribution and was the closest approximation to a normal distribution. The residual histograms from the AID488862 (**c**) and AID1224905 (**d**) data sets showed no evidence of being normally distributed. All the data sets contained thousands of compounds in the residual distributions. The “active” distribution extended to both sides of the “inactive” distribution and observations on both sides have a high likelihood of being active (**b**,**d**,**f**). Panels (b,d,f) may be a useful alternative to a receiver operating characteristic curve^7,29^.

The AID 1053175 screen for inhibitors of *E. Coli*, gave $\bar{x}$ = 0.6881 a.u. and *s* = 0.0414 a.u. with a histogram of mean values inconsistent with a normal distribution (Fig. 3a). The observed distribution may be bimodal or multimodal and/or skewed. The histogram of standard deviations, however, showed excellent correspondence to a single Gaussian process (Fig. 3b). A small number of compounds exhibited excess standard deviations. In the region with divergence from equation 1, 331 compounds were found with 194 (0.06% of the total) outside expectations of a single normally distributed process. This indicates that with the exception of 0.06% of all compounds any lack of correspondence to a normal distribution is due to compound activity variability and not from an inherently skewed statistical process.

The standard deviation estimated from a fit to the DSD (0.0296 a.u.) was slightly lower than obtained from the best fit normal distribution (*μ* = 0.6894 and *σ* = 0.0332 a.u). Both were lower than computed from all the mean values (*σ* = 0.0414 a.u.) indicating that many procedures for setting thresholds may be unintentionally excluding active compounds due to overestimation of the relevant standard deviation. The DSD provides a useful alternative.

In this case, the class of compounds having mean values below 0.590 (the *μ* − 3*σ* cut off) contains 2390 compounds of which 1905 are expected to be “active” and 485 “inactive”. This is higher than the number found in the original screen which used a somewhat different set of criteria for assigning “active” compounds.

Similar considerations were applied to the AID 488862 screen (Fig. 2c,d) for inhibitors of the prion protein 5′ UTR mRNA and the AID 1224905 screen (Fig. (2e,f) for small molecules disrupting the interaction between Gα_i_ and GIV (Gα-interacting vesicle-associated protein). The AID 488862 screen also reported the correlation cosine metric but did not use it in decision making (see SI section S.1). Both histograms of mean values exhibited some conformity to a normal distribution with additional broad asymmetric tails on both sides (Fig. 2c,e). The best fit normal distributions (AID 488862: *μ* = −12.28, *σ* = 13.10%; AID 1224905: *μ* = 103.6% and *σ* = 4.9) gave similar standard deviations to that obtained from the best fit DSD (AID 488862 *σ* = 14.3% & AID 1224905 *σ* = 5.2%) and both were lower than the standard deviations of the whole data sets. However, the distributions of standard deviations (Fig. 2d,f) showed less conformity with single normally distributed processes than did the screen for inhibitors of *E. Coli* (Fig. 2a,b). The AID 488862 data gave least agreement and 4620 compounds were observed with standard deviations greater than 2*σ* (28%) where only 1730 would be expected from a single normally distributed process. This may indicate a heteroscedastic process but due to the position of the mean (−12.281%) simple fluctuation scaling approaches to assess this were unsatisfactory. If the assumption that the inactive distribution was from a single normal distribution is correct, the value of *σ* (13.10%) from the best fit normal distribution is best for setting thresholds. When applying this assumption, the consequences of heteroscedasticity need to be appreciated (see SI section S.3) and the DSD can assist. These results make clear that a sub-population exists which belongs to a different process with excess variability (high standard deviation) and that the majority of measurements can be explained by paired trials sampling a single normal distribution.

### Residual distributions

As noted, presentations of “active” compound selection usually present well-spaced normal distributions or roughly equal size (see SI section S.3). To test the assumption that “active” compounds (those not belonging to the best fit normal distribution) follow normal distributions and to estimate their characteristics, the residual histograms were computed (Fig. 3a,c,e). Modelling the data in this manner also allows the ratio of “active” to “inactive” compounds to be computed for each position along the response axis (Fig. 3b,d,f). This approach was applied to the three screens considered previously (Fig. 2). The residual distribution from the AID 1053175 data contained ~27,000 compounds but did not show an obvious center (Fig. 3a). The maximum appeared approximately 1.5–1.7 *σ* below the center of the “inactive” distribution depending on whether the *σ* was from the best fit normal (1.5) or DSD (1.7). Based on the shape, the “true” center may be closer to the inactive distribution but in the absence of better models of “active” distributions and “inactive” behavior this remains speculative. The AID 488862 data set gave a nearly symmetric residual distribution (Fig. 3c) containing ~25,000 compounds having a maximum ~2.4*σ* below the center of the “inactive” distribution and no resemblance to a normal distribution. The AID 1224905 data gave a residual distribution (Fig. 3e) containing around 14,000 compounds with a maximum ~2*σ* above the center of the inactive distribution. The residuals showed no resemblance to a normal distribution. Consideration of the probability plots (Fig. 3b,d,f) suggests that “active” compounds may appear on both sides of the inactive distribution.

These results support three clear conclusions. 1) Compounds not contained in a single “inactive” distribution were not rare (~66,000 in these three data sets) and represented 6.7–8.2% of compounds tested. 2) “Active” and “inactive” distributions were not always well-spaced and a large number of “active” compounds were hidden under the wings of the “inactive” distributions. In the absence of better measurements, it is impossible to identify these compounds. 3) Compounds not contained in the “inactive” distribution do not resemble normal distributions and exhibit a variety of shapes. In the absence of knowledge of the position, scale, and shape of “active” compounds it is difficult to set reasonable thresholds. This pool of ~66,000 compounds represents an enormous opportunity currently lost in the noise of current methods.

### Follow up data

**Figure 4:**
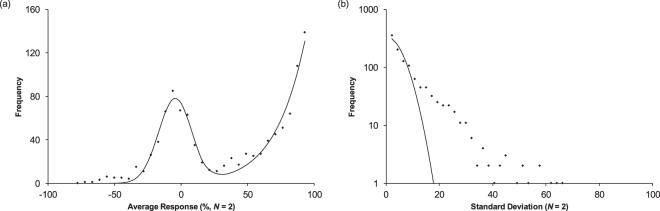
Histogram of mean values (**a**) and histogram of standard deviations (**b**) for the 504592 data set (1121 compounds with *N* = 2). The histogram of mean values (left panel) was fit to two distributions (solid line) to account for the bimodal behavior. The clear bimodal behaviour allows the high mean “active” group to be separated from other compounds. The distribution of standard deviations indicates enrichment of high variability compounds and less resemblance to a normally distributed process than the primary screen.

The AID 504592 screen was a follow up study of 1121 compounds assigned as “active” in the AID 488862 screen (Figs 2c,d and 3c,d). The follow on data set indicated that roughly 50% false positives were generated by the procedures applied to the primary screening data. In the original processing of the follow on screen (1121 compounds, 540 active, 525 inactive), three outcomes were indicated: i) if both measurements exceeded 50% it was declared “active”, if only one value was above 50% then the compound was deemed “inconclusive”, and if both values were below 50% it was declared “inactive”. A histogram of the average values (Fig. 4a) exhibited a peak showing reasonable correspondence to a normal distribution (*μ* = −4.5% and *σ* = 12.4) having a standard deviation close to that of the original screen. More importantly, this follow on screen had much poorer correspondence to a single normally distributed process and the best fit to a single DSD (Fig. 4b) gave a marginal fit with *s* = 7.37. The follow on screen was enriched in compounds exhibiting excess standard deviation (Fig. 4b). If a standard deviation >10% is considered as the threshold where the distribution of standard deviations begins to diverge from the expectations of a single process, a subgroup of 322 compounds are found (of which only 16 were assigned as active in the original study). Of these 255 are expected to be true high variability active compounds with 67 false positives. This high variability subgroup was heavily enriched and made up 23% of all compounds in the follow up study. If “active” is defined as a compound exhibiting either an average greater than *μ* +3*σ* (*μ* = −4.5% and *σ* = 12.4) and/or a standard deviation greater than 20%, a total of 721 compounds is found. Although unclear from the histograms, compounds deemed as “active” in the original assessment of the data set show a high degree of bias toward compounds with low standard deviations. This is due to the 100% upper bound in the measurements. These results add further support to the idea that a subpopulation of compounds exists exhibiting high variability. It also demonstrates that these compounds are enriched in this pool of compounds deemed active in the primary screen. In addition, data sets with an imposed upper or lower bound will deviate from simulations using widely held assumptions (see SI section S.3).

### Special cases: lognormally distributed data and indicators of bias

**Figure 5:**
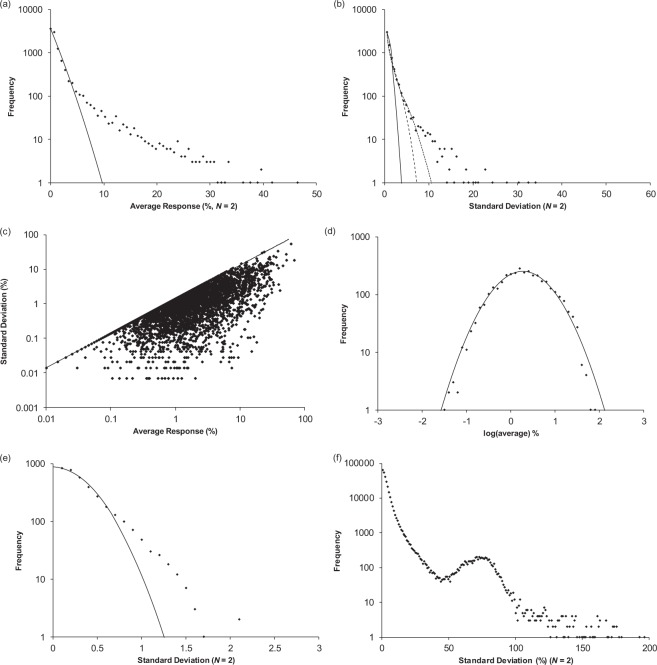
Histogram of means (**a**) and standard deviations (**b**) for the 540333 data set (10,240 compounds with *N* = 2). The histogram of mean values showed no signs of Gaussian behaviour (solid line). The histogram of standard deviations was fit to 1 (solid line), 2 (dashed line) and 3 (dotted line) distributions of standard deviations, none of which were satisfactory. The fluctuation scaling plot (**c**) represents the average and standard deviation obtained from 2 replicate measurements as a single point ($\bar{x}$, *s*). The line is an approximation to the upper bound in the relationship indicating some type of bias in the data set. The histograms of means (**d**) and standard deviations (**e**) showed reasonable correspondence to a log-normal distribution. (**f**) Histogram of standard deviations for the AID 651654 data set.

Two of the data sets were considered special cases due to signs of unintentional bias (AID 540333 and AID 651654) and lognormally distributed data (AID 540333). Histograms of the averages and standard deviations reported for the AID 540333 screen for inhibitors of the Dengue fever virus (10240 compounds; 318 active) exhibited no correspondence with a normal distribution (Fig. 5a) or a single normally distributed process (Fig. 5b). Attempts were made to introduce additional DSDs but these did not provide satisfactory results (Fig. 5b). A fluctuation scaling plot (Fig. 5c) of the measurements exhibited heteroscedasticity with the overall plot showing bias in the form of an upper level cut off. Further inspection of the data set revealed 3591 compounds that had zero average response, 2809 compounds had only a single non-zero measurement, and only 3840 out of 10240 compounds had two non-zero measurements. The bias in the fluctuation scaling plot may be due to an unspecified instrumental cut-off setting “outliers” to zero when they were found outside a particular range. Restricting the data to only the 3840 compounds having two valid non-zero measurements left a scaling plot still exhibiting signs of bias (not shown). Figure 5c suggested power law fluctuation scaling (equation 2) with an exponent near one, however due to the bias in the data set this is speculative. These observations aside, this data set was remarkable as the only one suggesting power law scaling law and was unique in this regard.

The data exhibited better correspondence to a log-normal distribution (Fig. 5d) and a log normally distributed process (Fig. 5e). From the lognormal histogram there was no evidence that any compounds could be assigned as active. Instead, there was evidence of under populated wings in the distribution, an effect ascribed to the previously noted bias in the data set. The data did not belong to a single log-normally distributed process (Fig. 5e); however, the shape of the histogram of standard deviations has been affected by the loss of 6760 compounds. If Fig. 5e is taken as correct, there were 446 compounds having log-standard deviation ≥0.7. Of this set, 248 are expected to be excess variability “active” and 198 false positives. At present there is no theory for how to assign hits in a heteroscedastic system following an exponential dispersion model. Were it not for the bias apparent in the data, this data set might have been an opportunity to test strategies.

The AID 540333 data set makes clear that the characteristics of the inactive distribution and the statistical process must be well understood to set meaningful thresholds (including for instrumental parameters and outliers). In the original screen, compounds were classed as active when the value was 3 *s* greater than the mean ($\bar{x}$ = 1.49; *s* = 3.92). These criteria probably resulted in 100% false positives.

The histogram of standard deviations (Fig. 5f) obtained from the AID 651654 screen for compounds causing lysis of *C. neoformans* (361009 compounds tested; 735 active) exhibited a prominent peak between 50–100%. Considering Fig. 1, it is clear that no combination of scaled DSDs based on any set of normal processes with *N* = 2 can produce such a shape. Normal distributions only show a peak in the DSD when *N* ≥ 3. It is also doubtful there are any reasonable alternatives to the normal distribution capable of producing such a shape. Similar to AID 540333, the AID 651654 data contained a large number of compounds (78,305) missing a second measurement. It is likely that the mechanism causing their absence introduced bias into the data set such that a section of the histogram of standard deviations was under-populated.

Both AID 540333 and AID 651654 showed signs of bias probably produced by a combination of missing data, “outlier” trapping, instrument saturation, and extreme value deletion. Whatever the causes, it resulted in under-populated wings of a log normal distribution in one study and an impossible histogram of standard deviations in another. This may be causing valuable lead compounds to be missed. The observed bias is almost assuredly unintentional but the mechanisms creating it need to be better understood.

### The distribution of standard deviations for N = 2, N = 3, and N = 4

**Figure 6:**
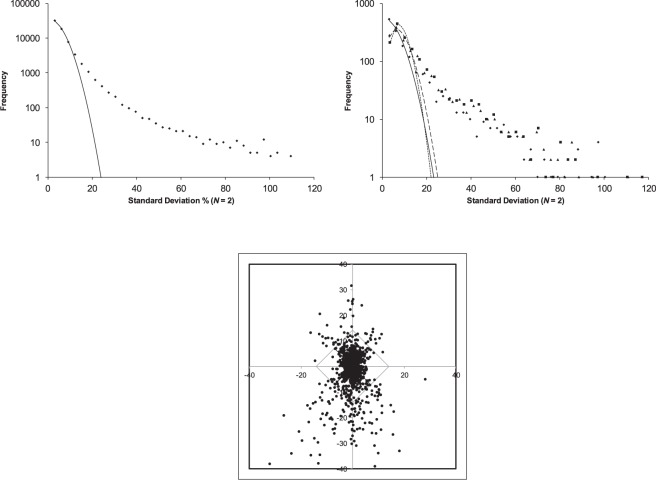
Histogram of standard deviations for the 66878 compounds measured with N = 2 under similar conditions (**a**), overlay histograms of standard deviations (**b**) for *N* = 2 (◆, fit solid line), *N* = 3 (▴, fit dashed line), and *N* = 4 (▪, fit dotted line), and a three dimensional representation of the *N* = 3 subset (**c**) with the three replicate measures plotted as (*x*, *y*, *z*).

A screen having a number of compounds measured 4 times was considered (AID 1053188). This cell based screen for inhibitors of CD40 signalling tested 83,073 compounds and assigned 780 as “active”. Within this set, 1512 compounds were measured four times; a further 1525 measured three times; 1233 had a single measurement; and the remainder were measured twice although 14,962 of these were measured at a higher concentration. Here the full set of 66,878 compounds measured twice at the same concentration were considered (Fig. 6a) along with the 1512 compounds whose behaviour could be compared by considering *N* = 2, *N* = 3, and *N* = 4 (Fig. 6b). In this comparison, the *N* = 2 set was the first two measurements, the *N* = 3 set was the first 3, and the *N* = 4 considered all 4 measurements. The recovered best fit standard deviations were 7.35%, 9.17%, 10.99%, and 10.18% for the *N* = 2 (66878 compounds), *N* = 2 (1512 compounds), *N* = 3 (1512 compounds), and *N* = 4 (1512 compounds) cases, respectively. Qualitatively, the predictions of equation 1 were observed with a maximum appearing in the distribution for *N* > 2 and the height of the maximum increasing with the number of measurements. All the histograms exhibited a long tail which was not affected by increasing *N*. The long tail of the distribution may be due to increased variance at larger values (Fig. 5c) possible suggesting a heteroscedastic measurement process.

### High variation active compounds

**Figure 7:**
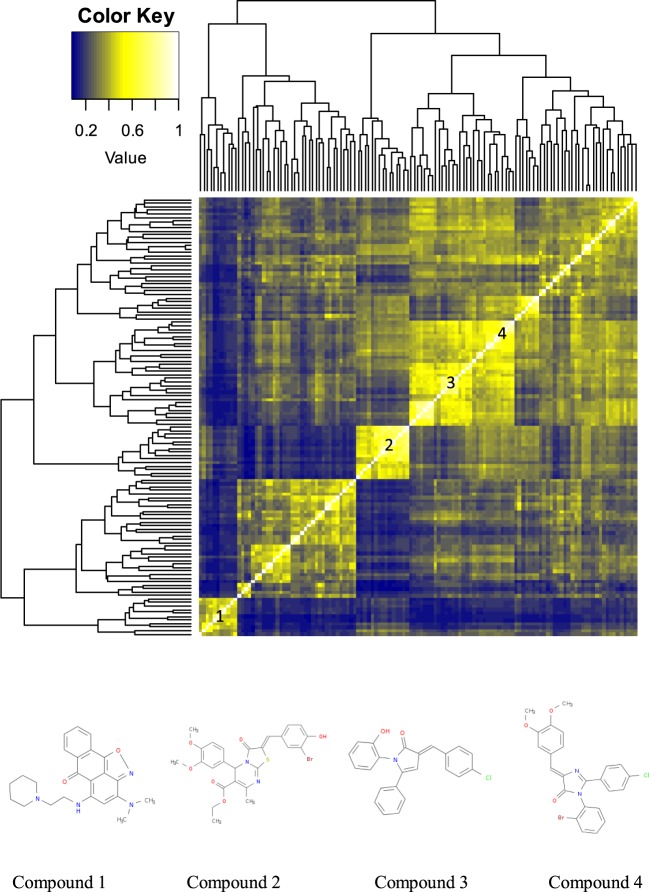
Heatmap with dendrograms generated from the four largest groups of compounds having Tanimoto values > 0.5. The numbers on the diagonal indicate the locations of compounds similar to compounds 1, 2, 3, and 4.

The compounds with high standard deviations were aggregated into a single set of 1189 compounds. To select this group a threshold of 3 standard deviations was set for inclusion resulting in 533, 484, 147, and 25 compounds being included from the AID 1224905, 488862, 1053175, and 540333 screens, respectively. These were clustered by Tanimoto distance^30,31^ with single connectivity using two approaches. In the first approach, the compound list was pre-sifted by binning them into groups based on Tanimoto values > 0.5. The four largest groups consisting of clusters of 55, 49, 11, and 10 compounds were then evaluated and presented as a heatmap with dendrogram (Fig. 7). Within this set, there were clearly defined compound groups with a high degree of structural similarity. Examples included: 11 compounds similar to the isoxazol-6-one (compound 1)^32,33^, 12 substituted ethyl-2-[(phenyl)methylidene]-5-(phenyl)-7-methyl-3-oxo-5H-[1,3]thiazolo[3,2-a]pyrimidine-6-carboxylates (compound 2), 13 substituted 5-phenylpyrrol-2-ones (compound 3), and eight substituted 3-(phenyl)-2-(phenyl)-5-[(phenyl)methylidene]imidazol-4-one compounds (compound 4). A sift of this subset found 72 (57.6%) considered to be PAINS indicating that the most structurally similar compounds are likely to be problematic for additional reasons^16,34–36^. In the second approach, all 1189 compounds were included (Figure S4) and of these 245 (21%) were classed as PAINS.

What is clear from both approaches is that many of the compounds showing high variability in four dissimilar HTS screens exhibit a high degree of structural similarity. It is unclear if the properties of these compounds make them inherently difficult to screen leading to highly variable results and/or all the screening methods have inherent heteroscedasticity and these are active compounds exhibiting increased variation. In either case, they deserve further scrutiny.

## Conclusion

This study has demonstrated the application of the DSD to large scale HTS measurements consisting of a large number of samples measured a small number of times. The DSD can provide insight when there is variation in sample mean values such that a histogram of means produces a range of distribution shapes. If the data sets examined here are representative of HTS generally, a number of conclusions apply.

1. The view of discrimination of “active” compounds as the problem of separating two well-spaced normal distributions is false, at least most of the time. The “active” distribution may have its center under the wings of a main distribution of “inactive” compounds and its tails may extend to both sides of the “inactive” distribution.
2. There is no evidence that “active” distributions are normally distributed or follow other well known statistical models. The residual plots provide considerable evidence to the contrary. This makes chemical sense because chemical “activity” as currently understood depends on compound structure, dynamics, and shape relative to some target structure, dynamics, and shape. Variation in chemical structures is discrete in nature and fundamentally discontinuous. As such, a normal distribution will always be an approximation.
3. There appears to be a subset of compounds, designated here as high variability “active”, that are not well served by current HTS methods. Because they have high variability, it is also likely they have mean values with large magnitude (see Figs 5c and 6c). It is important to try to identify these compounds and if multiple screens show particular classes of compounds to be in this category, they should be further investigated and if necessary alternative methods developed. Based on the current work, many of these compounds were PAINS.
4. There are a large number of “active” compounds hidden under the measurement noise of “inactive” compounds and as a result not being identified (see Fig. 3a,c,e). This suggests the current state of the art in measurement science is fundamentally limiting the discovery of drugs, at least in publicly available data sets. Better measurements are needed. In the meantime, practitioners of HTS should reconsider how they class compounds as “active”. The distribution of compound measurements is not a good way to characterise *σ* for the purpose of setting thresholds. If a large number of “active” compounds are present and they lie close to the “inactive” distribution, defining “active” by expected true positive to total compound ratio in an interval is a better approach both for deciding which compounds to study further and for providing a more nuanced set of inputs for machine learning algorithms and cheminformatics^37,38^.

Overall, this study makes clear that many lead compounds for future drugs may be lost in current HTS. With a high degree of likelihood the following are clear: (i) compounds are getting lost due to biased decision making (e.g. missing data, excluded results, arbitrary thresholds, etc.); (ii) compounds are getting lost due to not being able to separate measurement error from variability in compound response; (iii) compounds are not being detected due to noisy measurements; and (iv) compounds are getting lost because they are inherently difficult to screen.

This represents considerable opportunity. As an example, in the search for new antibiotics options are limited. Discovery of inexpensive weakly antibiotic compounds sufficient for use to promote animal growth could remove the incentive to use more potent ones for this purpose. Chemists able to discover the weakest and cheapest compounds suitable for this purpose would be doing a great service. Such compounds are not going to be found by current methods and represent a useful challenge. Finally, the interest of synthetic chemists and data miners in HTS outcomes are not well served by a binary “active”/“inactive” designation. A marginally active compound for a chemist is a synthetic challenge and opportunity.

## Materials and Methods

The following data sets were obtained via PubCHEM^39,40^: AID 488862, AID 504592, AID 540333, AID 1053175, AID 1224905, AID 1053188, and AID 651654. These screens were selected for replicate measurements (*N* ≥ 2), the size of the reported compound library, to represent a range of assay formats, and to illustrate a range of behaviours. As a group, they included 4 cell based assays, 2 organism assays, and 1 biochemical assay. There were 3 studies with gene reporters, one cell viability assay, one displacement assay, one functional assay, and one coupled enzyme assay. All but one was measured using some form of luminescence with the exception using absorbance. One of the data sets (AID 504592) was a follow up study to a primary screen (AID 488862). For context, a PubCHEM search for assays restricted to “screening” returned 1027 results. Roughly half of these fell into the categories “cell-based” (281) and “biochemical” (215) with 481 apparently unclassified. Related searches of the ChemBL^41,42^ assay data base for “assay” returned 185,885 records. Restricting this to “HTS” yielded 1216 data sets. Of these, 827 were from PubChem, 302 from the Scientific Literature, and the remainder from GSK, Novartis, and BindingDB^43^. The 1216 records were classified by assay format: 208 were cell-based, 143 were organism based, 18 were biochemical, 10 were small molecule physicochemical, 7 were tissue based, and the remainder (830) unclassified. Separately they were also classified by assay type with most being considered as either functional (962) or binding assays (228). Other routes into PubCHEM and ChemBL will give different numbers of records, however, these data give a rough picture of the importance of different assay types in publicly available HTS data sets.

AID 488862 was a primary screen carried out by the Broad Institute for compounds inhibiting the prion protein 5′ UTR mRNA using a *cell based* (H4), *reporter gene* assay read out by *bioluminescence*^44^. The data set was from 2010 and covers 335,011 compounds were screened with 1169 deemed active. AID 504592 was a follow on screen of compounds assigned as active from AID 488862 using the luciferase reporter and luminescent readout. The data set is from 2011 and included 1,122 compounds with 540 deemed active.

AID 540333 was a screen carried out by the Southern Research Institute Specialized Bio-containment Screening Center for inhibitors of the Dengue fever virus using cells (BHK21 (C-13)) treated with Dengue virus 2 which were subsequently read out with a luminescent cell viability assay^45^. The PubCHEM bioassay annotations classed this as a *cell based*, *cytotoxicity* assay, read with *luminescence*. The data set is from 2011 and reports results from 10,240 compounds with 318 deemed active.

AID 1053175 was a screen carried out by the ICCB-Longwood Screening Facility for molecules inhibiting the growth of *E. coli* using measurements of absorbance at 600 nm on cultures^46,47^. Bioassay annotations were missing from the PubCHEM description, however, based on the protocol this was classed as an *organism* based *functional* assay read using light *absorbance*. The data set from 2016 provides results from 329,176 compounds with 702 deemed active.

AID 1224905 employed a polarization assay involving displacement of a short FITC labelled GIV peptide to screen for small molecules disrupting the interaction between Gα_i_ and GIV (Gα-interacting vesicle-associated protein)^48^. Bioassay annotations were missing from the PubCHEM description, however, based on the protocol this was classed as a *biochemical* displacement assay read using *fluorescence polarization*. The study was carried out by the ICCB-Longwood Screening Facility. The data set is from 2017 and includes 206,873 compounds with 879 deemed active.

AID 1053188 was carried out by the Broad Institute using a cell based luciferase reporter assay read out by luminescence to screen for inhibitors of the cell surface receptor CD40. Bioassay annotations were missing from the PubCHEM description, however, based on the protocol this was classed as a *cell-based* (BL2)*, gene reporter* assay read using *bioluminescence*. The data set from 2015 reports the results from 83,073 compounds with 780 deemed active. This screen was included due to 1512 compounds having 4 replicates.

AID 651654 was carried out by the Broad Institute to search for anti-cryptococcal compounds detected by release of adenylate kinase. The PubCHEM bioassay annotations classed this as an *organism based*, *coupled enzyme activity* assay, read with *chemiluminescence*. The data set from 2012 reports the results from 361,009 compounds with 735 deemed active.

### Statistics and cheminformatics

Descriptive statistics, histograms, and distribution fitting were done using MS Excel. Structural clustering analysis and visualization were done using R (Version 3.4.4)^49^ with the ChemMineR (Version 2.30.2) package^50–52^. Compounds were filtered for PAINS using FAF-Drugs4^36^ on the RBPS Web Portal^34,35^.

## Electronic supplementary material

Supplementary Information for The Distribution of Standard Deviations Applied to High Throughput Screening

## Data Availability

All the data analysed here are freely available via PubChem and may be obtained by following the links provided. AID 488862: https://pubchem.ncbi.nlm.nih.gov/bioassay/488862; AID 504592: https://pubchem.ncbi.nlm.nih.gov/bioassay/504592; AID 540333: https://pubchem.ncbi.nlm.nih.gov/bioassay/540333; AID 1053175: https://pubchem.ncbi.nlm.nih.gov/bioassay/1053175; AID 1224905: https://pubchem.ncbi.nlm.nih.gov/bioassay/1224905; AID 1053188: https://pubchem.ncbi.nlm.nih.gov/bioassay/1053188; AID 651654: https://pubchem.ncbi.nlm.nih.gov/bioassay/651654.
