# Supplementary material for: The Distribution of Standard Deviations Applied to High Throughput Screening
Source: Sci Rep. 2019 Feb 4;9:1268. doi: 10.1038/s41598-018-36722-4 (PMC6361996; doi:10.1038/s41598-018-36722-4)
Supplement: Supplementary file 1 — Supplementary Information for The Distribution of Standard Deviations Applied to High Throughput Screening [file 41598_2018_36722_MOESM1_ESM.pdf]

Supplementary Information for The Distribution of Standard Deviations Applied to High Throughput Screening

Quentin S. Hanley*

School of Science and Technology
Nottingham Trent University
Clifton Lane
Nottingham NG11 8NS
United Kingdom
quentin.hanley@ntu.ac.uk
Telephone: +44 (0) 115 848 3536

*** Corresponding author:** Q. S. Hanley

S.1: The Reproducibility Cosine Transform

The reproducibility cosine transform (RCT)[1] is systematically reported in a subset of screens on PUBCHEM[2,3] (examples include: AID 1662 (a screen for inhibitors of streptokinase expression),[4] AID 1979 (a screen for inhibitors of *C. Albicans*),[5] AID 2099 (a screen for inhibitors of histone demethylase GASC-1),[6] AID 2241 (a screen for inhibitors of the A1 anti-apoptotic factor),[7] AID 588549 (a screen for inhibitors of the tuberculosis fatty acyl-AMP ligase FadD28),[8] AID743287 (a screen for activators of glucose dependent insulin secretion),[9] and AID 1053188 (a screen for inhibitors of CD40 signaling)[10]). This is not an exhaustive list and RCT is mentioned in a number of papers reporting screening results.[11-13] As it appears in the PUBCHEM screens, this parameter is the absolute value of the cosine similarity of a set of numbers, **A**, with the *N* dimensional unit vector **B** (Equation S.1) and ranges from 0 to 1.

$$RCT = \frac{|\mathbf{A}\cdot\mathbf{B}|}{\|\mathbf{A}\|\|\mathbf{B}\|} = \frac{\left|\sum_{i=1}^{N} A_i B_i\right|}{\sqrt{\sum_{i=1}^{N} A_i^2}\sqrt{\sum_{i=1}^{N} B_i^2}} \quad \text{(S.1)}$$

In many descriptions, a value of 1 is considered to be perfect reproducibility. Considering Figure 1 of the main text, it is clear that a large proportion of RCT values are expected to be near one as the distribution of standard deviations is maximal near zero for $N = 2$. This can be verified using pairs of normally distributed random numbers (Figure S.1). For N=2, values near one are more likely to be fortuitous rather than a meaningful indicator of data quality. As N gets larger, the likelihood decreases, but with even a modest offset of the average response from zero RCT shows an increasing tendency to exhibit values near 1. RCT is sensitive to offsets (Figure S.1). As such, it is of limited utility in assessing reproducibility in a meaningful way and where used explicitly in some screens with $N = 2$ it does not appear to add anything to composite z-score.[11]

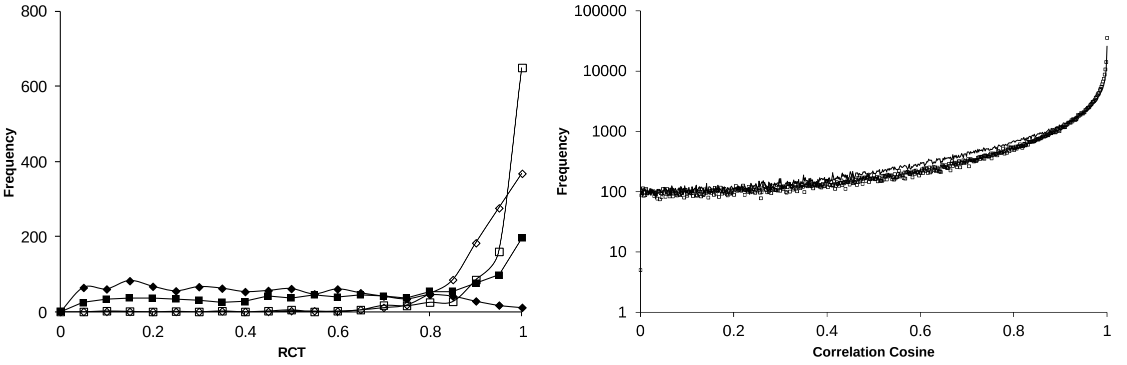

Figure S.1: RCT computed for 1000 trials of normally distributed random numbers with μ = 0, σ = 1 (solid symbols) with μ = 2, σ = 1 (open symbols) (left panel). The diamonds represent N=4 and the squares represent N=2. Histogram of RCT values from the AID488862 data set (right panel) compared to an RCT histogram 335011 pairs of random numbers with μ = -12.3 and σ = 10.8 %. The open squares are from the histogram of measured RCT values.

The AID 488862 data set reported reproducibility values using the correlation cosine. For comparison, a set of 335011 pairs of normally distributed random numbers were generated having the mean from the best fit normal distribution and the standard deviation obtained

from the best fit to the distribution of standard deviations. The correlation cosine was computed for each pair and a histogram generated and compared to the histogram observed in the screen (Figure S.1, right panel). Nearly all the variation in *RCT* can be explained by paired trials sampling a single normal distribution. The correspondence is very good indicating that nearly all of the behaviour of the correlation cosine in this data set is consistent with random variations from a single normal distribution and not indicative of the quality of any particular measurement. For the vast majority of the 335011 compounds measurements, high *RCT* is fortuitous and irrelevant to deciding measurement quality. 3) The use of the correlation cosine as an indicator of reproducibility can be mostly explained by normally distributed random numbers. This was tested for $N = 2$ and it is likely this is generally the case. As such, the distribution of RCT values observed in a large scale screen is well modelled by a random number generator. Any particular high or low value is fortuitous and has no interpretive meaning.

S.2 Double or Multiple Thresholds

The use of double thresholds is nearly universal in PUBCHEM primary screens where $N = 2$. This practice requires each of two or more replicates to meet some threshold value as a criterion for assigning a compound as active. This is the case for most of the data sets considered in this study and is an effective way to select for compounds that have a strong activity in an assay. However, for future cheminformatics studies to better understand drug activity and to discover weaker drugs or drugs from marginal assays, the strategy may be counterproductive. This is particularly true in the absence of a clear understanding of the variability of an assay under screening conditions and the behaviour of "inactive" compounds. As an example, consider trying to discriminate active compounds from normally distributed

random numbers having μ = 0 and σ = 1. Single trials are expected to have 95% of values between ±1.96σ. Trials with N = 2, are expected to have 95% of points lying within a circle centered on the origin having a radius of 2.45σ (Figure S.2). The application of two one-tailed thresholds representing single trials at 95% confidence (e.g. +1.96σ) transforms a polar co-ordinate problem into a Cartesian system. A section of the co-ordinate system is defined by this procedure in the upper right of figure S.2 with its vertex touching a circle centered on the origin having radius 2.77σ. It needs to be remembered that in the absence of "active" compounds, ***all*** observations represent "inactive" compounds no matter where they are found or what thresholds are set.

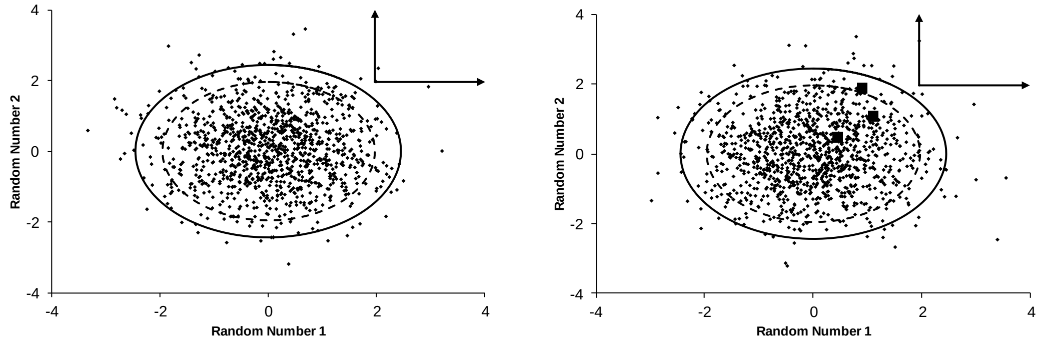

Figure S.2: Pairs of normally distributed random numbers (μ = 0, σ = 1). For single numbers, 95% are expected to lie inside of 1.96σ (dashed line). For pairs of numbers, 95% are expected to lie inside a circle of radius 2.45σ (solid line). Requiring both values to exceed a threshold, representing "95% confidence" in both x and y directions defines a region (arrows upper right) extending from (1.96, 1.96) outward. This conforms to a subset of the region outside a circle of radius 2.77σ. The left panel consists of only "inactive" compounds. The right panel includes 0.3% active compounds (large squares) from a distribution with (μ = 0.5, σ = 1). The "actives" were undetectable.

This can be generalized to any N: 95% of observations are expected to lie within the N-sphere of radius equal to the square root of the cumulative chi-square distribution for a particular N (e.g. 1.96, 2.45, 2.80, 3.08, 3.33, ... for N = 1, 2, 3, 4, 5, ...).

Both approaches – RCT and double thresholds – could be replaced by polar distance and angle and search for compounds with results above a given polar distance and within a specified angle range bracketing 45 degrees. This allows potentially active compounds to be discriminated from inactive compounds nominally inside an N-sphere and the probability of finding normally distributed observations is comparatively easier to compute. For example when $N = 2$, only 0.06% of observations are expected with polar distance > 2.45σ and within $\pm 2^{o}$ of 45°.

S.3: HTS active assignment and normal distributions with heteroscedasticity.

Traditional presentations of estimates of false positive and false negative decisions usually are presented using well-spaced homoscedastic Gaussian distributions of equal size. Although these are statistically correct in presentation, the hypothesis of well-spaced homoscedastic Gaussian distributions of equal size needs greater testing. Understanding the impact of variably spaced heteroscedastic distributions of unequal size is critical for setting thresholds for detecting "active" compounds. Considerable additional complexity is possible (e.g. skewed distributions, log-normal distributions, etc), however, cases were simulated with two normal distributions having "active" and "inactive" distributions with equivalent standard deviations, with an "active" distribution having a 50% larger standard deviation, and with an "active" distribution having a 50% lower standard deviation. The "active" distribution was assumed to be 1, 2, and 4 standard deviations away from the "inactive" distribution. Cases where the "active" distribution was equal to the "inactive" distribution were compared to a more realistic set of unequal distributions based on the data from the screens investigated in this paper (99.7% "inactive" and 0.3% "active). These are shown in Figure S3. Several points are worth noting. 1) Depending on the conditions modeled, moving further away from the inactive distribution may lower the likelihood of finding an active compound. It can be advantageous for the "active" distribution to have a higher standard deviation than the

“inactive” distribution. When the opposite is the case and the distributions are close, it can be impossible to detect an active compound with a reasonable probability. Under these conditions observations far away from the center of the “inactive” distribution are highly likely to be from the “inactive” distribution. 2) If there is heteroscedasticity and the “active” distribution has greater standard deviation, then “active” compounds may be found with reasonable probability on either side of the “inactive” distribution. 3) In the case of realistically populated distributions, observations may need to be 3, 4, 6, or more standard deviations away before the likelihood that a compound is active reaches 50% or greater.

The simulations make clear that conventional presentations (c.f Malo Figure 5)[14] used to explain assignment of compounds to categories of active and inactive using well separated homoscedastic distributions of equal size will lead to incorrect ideas about the best locations to find active compounds and how to set thresholds. Under some assumptions it is impossible to locate “active” compounds. By imposing upper bounds (e.g 100% activity) this can force unfavorable heteroscedasticity (active standard deviation lower than the inactive standard deviation) and make detection of compounds more difficult.

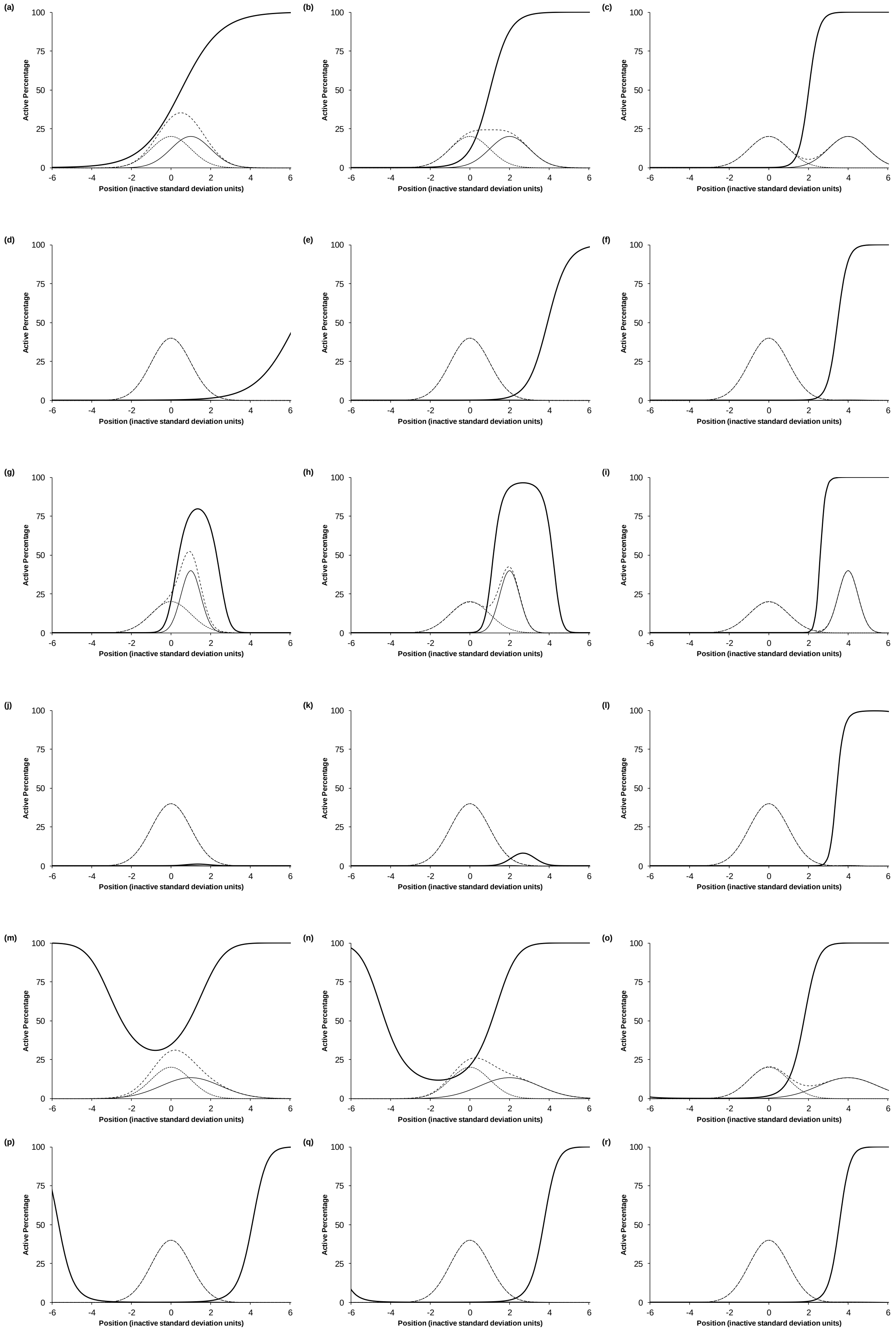

Figure S3: Simulations of Gaussian distributions separated by 1, 2, and 4 standard deviations when the relative size of the distribution varies. In all cases the heavy solid line represents the fraction of the total observations expected to be active expressed as a percentage. The long dashed line is the sum of the “inactive” (dotted line) and the “active” distribution (light solid

line). Panels (a-c) show equal sized homoscedastic distributions spaced 1, 2, and 4 standard deviations apart. Panels (d-f) show the unequal distributions (99.7 % “inactive” and 0.3% “active”). Panels (g-i) show equal sized heteroscedastic distributions spaced 1, 2, and 4 standard deviations apart in which the “active” distribution has a standard deviation 50% smaller than the “inactive” distribution. Panels (j-l) show the equivalent except with unequal distributions (99.7 % “inactive” and 0.3% “active”). Panels (m-o) show equal sized heteroscedastic distributions spaced 1, 2, and 4 standard deviations apart in which the “active” distribution has a standard deviation 50% larger than the “inactive” distribution. Panels (p-r) show the equivalent except with unequal distributions (99.7 % “inactive” and 0.3% “active”).

S.4: Heatmap and dendrogram of all 1189 high variability compounds.

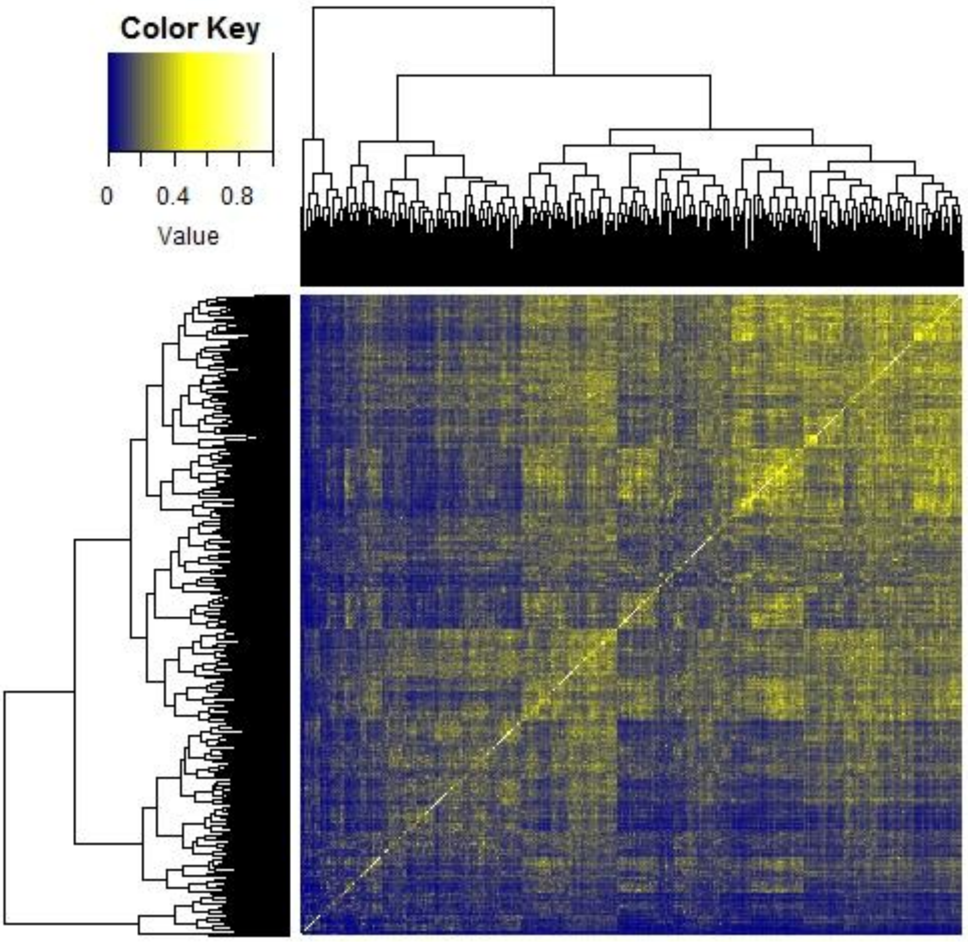

Figure S4: Heatmap with dendrograms generated from all 1189 high variability “active” compounds.

References:

1 Seiler, K. P. *et al.* ChemBank: a small-molecule screening and cheminformatics resource database. *Nucleic acids research* **36**, D351-D359 (2007).

2 Butkiewicz, M., Wang, Y. & Bryant, S. High-Throughput Screening Assay Datasets from the PubChem Database. *Chem Inform* **3**, 1 (2017).
3 Kim, S. *et al.* PubChem substance and compound databases. *Nucleic acids research* **44**, D1202-D1213 (2015).
4 Sun, H. *et al.* Plasminogen is a critical host pathogenicity factor for group A streptococcal infection. *Science* **305**, 1283-1286 (2004).
5 Redding, S. *et al.* Resistance of Candida albicans to fluconazole during treatment of oropharyngeal candidiasis in a patient with AIDS: documentation by in vitro susceptibility testing and DNA subtype analysis. *Clinical Infectious Diseases* **18**, 240-242 (1994).
6 Cloos, P. A. *et al.* The putative oncogene GASC1 demethylates tri-and dimethylated lysine 9 on histone H3. *Nature* **442**, 307 (2006).
7 Zong, W.-X., Edelstein, L. C., Chen, C., Bash, J. & Gélinas, C. The prosurvival Bcl-2 homolog Bfl-1/A1 is a direct transcriptional target of NF-κB that blocks TNFα-induced apoptosis. *Genes & development* **13**, 382-387 (1999).
8 Grimes, K. D. & Aldrich, C. C. A high-throughput screening fluorescence polarization assay for fatty acid adenylating enzymes in Mycobacterium tuberculosis. *Analytical biochemistry* **417**, 264-273 (2011).
9 Burns, S. M. *et al.* High-throughput luminescent reporter of insulin secretion for discovering regulators of pancreatic Beta-cell function. *Cell metabolism* **21**, 126-137 (2015).
10 Li, G. *et al.* Human genetics in rheumatoid arthritis guides a high-throughput drug screen of the CD40 signaling pathway. *PLoS genetics* **9**, e1003487 (2013).
11 Wierenga, K. J., Lai, K., Buchwald, P. & Tang, M. High-throughput screening for human galactokinase inhibitors. *Journal of biomolecular screening* **13**, 415-423 (2008).
12 Demirbas, D. *et al.* A yeast-based chemical screen identifies a PDE inhibitor that elevates steroidogenesis in mouse Leydig cells via PDE8 and PDE4 inhibition. *PLoS ONE* **8**, e71279 (2013).
13 de Medeiros, A. S. *et al.* Identification and characterization of a potent and biologically-active PDE4/7 inhibitor via fission yeast-based assays. *Cellular signalling* **40**, 73-80 (2017).
14 Malo, N., Hanley, J. A., Cerquozzi, S., Pelletier, J. & Nadon, R. Statistical practice in high-throughput screening data analysis. *Nature biotechnology* **24**, 167 (2006).
